# Testing distance characteristics and reference values for ice-hockey straight sprint speed and acceleration. A systematic review and meta-analyses

**DOI:** 10.5114/biolsport.2023.122479

**Published:** 2023-02-01

**Authors:** Petr Stastny, Martin Musalek, Robert Roczniok, Daniel Cleather, Dominik Novak, Michal Vagner

**Affiliations:** 1 Department of Sport Games, Faculty of Physical Education and Sport, Charles University in Prague; 2 Department of Sports Theory, The Jerzy Kukuczka Academy of Physical Education, Katowice, Poland; 3 St. Mary’s University, Twickenham, School of Sport, Health and Applied Science

**Keywords:** Anaerobic, Exercise, Condition, Testing, Sport training

## Abstract

Ice-hockey requires high acceleration and speed sprint abilities, but it is unclear what the distance characteristic is for measuring these capabilities. Therefore, this systematic meta-analysis aims to summarize the sprint reference values for different sprint distances and suggest the appropriate use of ice-hockey straight sprint testing protocols. A total of 60 studies with a pooled sample of 2254 males and 398 females aged 11–37 years were included. However, the pooled data for women was not large enough to permit statistical analysis. The sprint distance used for measuring the reported acceleration and speed was between 4–48 m. Increased test distance was positively associated with increased speed (r = 0.70) and negatively with average acceleration (r = -0.87). Forward skating sprint speed increases with the measured distance up to 26 m and do not differ much from longer distance tests, while acceleration decreases with a drop below 3 m/s at distances 15 m and longer. The highest acceleration (5.89 m/s^2^ peak, 3.31 m/s^2^ average) was achieved in the shortest distances up to 7 m which significantly differs from 8–14 m tests. The highest speed (8.1 m/s peak, 6.76 m/s average) has been recorded between 26–39 m; therefore, distances over 39 m are not necessary to achieve maximum speed. Considering match demands and most reported test distances, 6.1 m is the recommended distance for peak acceleration and 30 m for peak speed. The sprint time, acceleration, and speed of each individual and the number of skating strides should be reported in future studies.

## INTRODUCTION

Because of the number and quickness of game situation changes, ice-hockey is considered to be one of the sports with the highest requirement for speed, rapid changes of direction, cognition, and decision making [1, 2]. All of these factors are limited by the speed of motion, or the ability to accelerate and decelerate. Since acceleration and speed ability are critical factors to successful elite ice-hockey performance, they have been investigated in populations at various training levels [2–6]. However, the normative standards for skating acceleration and sprint ability are not well described in the current literature, as it is done for the sprint in running [7]. This is in turn aggravated by the use of different testing distances across studies. On the other hand, there is enough data reported in terms of the distance-time series to summarize general patterns in skating acceleration and velocity.

Among the previous studies, skating acceleration has been reported in distances ranging from 4 m [8] up to 20 m [9], or alternately in terms of the distance achieved in the first four or seven starting strides [10, 11]. Similar inconsistencies are evident in terms of speed, where the distance considered ranges between 25 [12] and 48 m [13]. This inconsistency aggravates the comparison between studies and sprint conditions since hockey locomotion is based on the propulsion-gliding mechanism [14]. If the sprint distance is shorter, the skaters are less reliant on the gliding phase, whereas when the distance is longer and closer to the length of the hockey rink (60 m), it is difficult to perform a full-speed effort to the finish due to the risk of collision with the barrier. Moreover, it has been shown that the initial acceleration phase and wide of the skating stride are more important to achieve maximal skating speed over a 30 m distance than step-by-step acceleration during the final stages of the sprint [15]. Another testing complication is the differences between skating to running sprints because it is not suitable to simply predict the velocity–time curve with a bi-exponential function which is valid for running [7, 16]. For instance, Perez applied Samozino´s method to the 30 m running and 40 m skating sprints, finding no correlation between running and skating in theoretical maximal velocity, theoretical maximal force, and 5 m acceleration time [17]. At the same time, total running and skating time were highly correlated (r = 0.80) [17], and 40 m skating sprint time had a moderate correlation [17]. Although the Samozino´s method provides sufficient reliability in skating [18] and correlates to some off-ice values [17] or resisted sprint values [19] its use has limits in evaluating ice friction [20] and lateral movement during skating strides [15].

High-intensity skating is a key factor in match performance and elite players skate at a speed above 17 km/h for at least 70 m each playing shift [21] and for about 2000 m per match [22]. This requirement varies by playing position and also between performance levels [23], which underlines its importance as a performance differentiating factor. However, the skating speed achieved depends also on the distance covered, where the skating slide helps to maintain the velocity, which is developed using a stride-by-stride acceleration strategy up to maximal speed [24]. Moreover, the skating start differs between performance levels in the knee, hip, and ankle kinematics [25], and in the skating economy [26]. Therefore, the distance to measure maximum acceleration and speed might differ between players but should be related to the expected stride span for the standard skating technique.

There are several approaches to identifying skating acceleration. Four stride accelerations cover 4 to 6 m distance [10] and are important for puck battles and spatial tactics. As the distance covered increases for each skating stride, the seven stride accelerations represent about 13.5 m distance [11] which is the distance from the center of the rink to the side barrier or the shortest distance between offence and defense zones. For longer distances skating should be performed using the full stride length but keeping stride by stride acceleration to achieve maximal skating speed [15] with stride frequency about 1.6 strides per second [27] and skating efficiency about 2.9 strides per m^-1^ · s^-1^ [28]. However, there is currently no comparison as to whether a distance of 20 m is enough to achieve maximum speed at full stride length, or whether the testing speed for 48 m represents the highest speed at a practically meaningful distance. Since the purpose of sprint testing is to compare different performance and age category groups, the uncertain distances for acceleration and speed testing impede the interpretation for the purposes of inter-individual or intra-individual condition evaluation and training recommendations.

As inconsistent distances are used for ice-hockey sprints, the aim of this systematic and meta-analysis is to summarize the sprint reference acceleration and speed values for different sprint distances and suggest the appropriate use of ice-hockey straight sprint testing. We hypothesize that the shortest acceleration distances of up to 7 m and the most used 30 m distance for speed testing are optimal for maximum acceleration and speed measures.

## MATERIALS AND METHODS

This review was performed in accordance with the PRISMA 2022 statement [29] adaptation for sport science [30] and registered with International Platform of Registered Systematic Review and Meta-analysis Protocols (INPLASY) on 03.05.2022 [no INPLASY202250010, DOI: 10.37766/inplasy2022.5.0010 316637]. An initial search in PubMed, Scopus, Web of Science, ProQuest, SPORTDiscus was performed on May 14 2022 using the combined terms “ice hockey OR “Ice-hockey” for all searched databases. Searches were limited to articles in English published in peer-reviewed journals since 1985. The database reports were uploaded to EndNote X9, duplicates were automatically removed, and then articles were screened for eligibility. Following the title and abstract screening, the remaining records were screened manually using the same PICOS (Population, Intervention, Comparison, Outcomes and Study design) strategy as for the database search and exclusion criteria. The reference lists of the selected full text articles and reviews were also examined to find further eligible articles.

### Eligibility criteria

The title and abstract screening was done by two researchers (PS and RR) who selected a set of articles for full text screening, where the inclusion criteria were: 1) male or female ice-hockey players; 2) any cross-sectional or intervention study; 3) tests of ice-hockey sprinting over any distance or any battery of conditioning tests that included straight-line sprints; and, 4) results reported straight-line sprint distance, speed, time, or acceleration. In the case of disagreement between the evaluating authors, the final decision was made by a third author (MV). The full text screening exclusion criteria were: 1) if the article was not in English; 2) the testing did not include straight-line sprinting; 3) the reported values did not include data distribution; 4) the study reported only maximum speed without skating time or average speed; 5) the end of the sprint was defined by the point the player stopped sprinting; 6) the measurement was made with a stopwatch; and, 7) the study had bias in item 3 or 7 according to JBI (Joanna Briggs Institute) Critical Appraisal Check-list for Analytical Cross Sectional Studies (Supplementary material 1). The maximum speed test was not included due to the uncertain velocity conditions at beginning of testing distance.

### Data extraction

Participant descriptions, test parameters and test results (mean and SD or other description of data distribution) were extracted to an Excel sheet separately by two researchers (PS, DN), where the test results were sorted by sprint distance, and age category. The extracted data describing sprint times (s) were transformed to average velocity (m/s) and average acceleration (m/s^2^) for the sprint distance.

### Statistical analyses

The average acceleration and velocity values were pooled by distance ranges of 0–7 m, 8–14 m, 15–25 m, 26–32 m, 33–39 m and 40–48 m. The normality and equality of variance (Bartlett’s test) of the original data was checked using the reported distribution in the original articles and weighted means with confidence intervals and standard error were calculated for each distance range. In the case of acceleration, equality of variances was violated. Therefore, we used Welch’s One Way Analysis of Variance (ANOVA) for unequal variances [31], followed by the Games-Howell post hoc test for multiple comparisons. For the speed analysis, we used One Way ANOVA with Tukey’s post hoc test where variances at each distance range met the equality requirement [31].

Other statistics have been done without pooling to distance ranges. The Kendall rank correlation coefficient (τ) and regression coefficient was used to express the relation between sprint distance, average velocity and acceleration. The players age correlation was included due to possible mediation to speed and acceleration parameters. Regression functions with 95% confidence intervals were used to describe the dependence of velocity and acceleration on the skating distance. All statistical analyses were done in STATISTICA software (TIBCO, Palo Alto, CA, USA) with R software 3.2.1 integration using an a priori significance level of p < 0.05.

## RESULTS

**FIG. 1:**
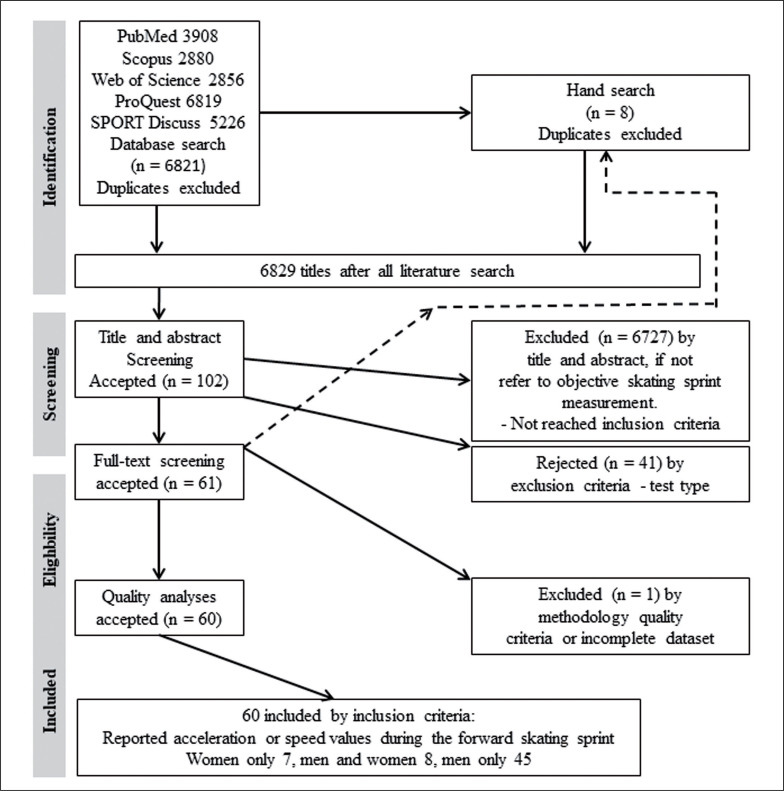
The flow chart of the systematic search for articles including ice-hockey forward sprint or acceleration data.

The database search resulted in 6821 titles after removing duplicates and 8 studies were added from the search of reference lists (Figure 1). Title and abstract screening identified 102 studies for full text screening. Of these, 41 studies were excluded due to data content and 1 study was excluded based on the quality criteria. Ultimately, 60 studies met all eligibility criteria and were included in the statistical analyses, of which 45 studies considered men only, 7 considered women only, and 8 studies included both genders (Table 1). The study of Nigg [32] included one female pooled into male group, therefore this study was presented as data on males. The average score of the JBI checklist for the articles included in this review was 81 ± 10%, ranging from 75% to 100% with 96% observed agreement between the two evaluators (PS, MV).

**TABLE 1:** The basic characteristic of selected studies on ice-hockey sprint performance.

Author	Participants n, gender, age (y), height (cm), BM (kg); specificity	Reported tests	Aim of the study	Main finding
Allisse et. al. 2019 [28]	24, male elite, 11-17 y, younger 150.4 ± 4.1 cm, 41.2 ± 7.3 kg; older 170.0 ± 13.2 cm, 68.4 ± 26.8 kg 11 pee-wee, 8 bantam 5 midget players from; Special study Saguenay, Quebec, CA	30.5 m sprint	To evaluate the robustness of equations to predict the oxygen requirement during different skating circumstances commonly found in ice hockey game situations (skating forward, backward, with and without controlling a puck, during cornering and stops and starts).	The skating execution time alone is a bad estimator of oxygen uptake. Age proved to be a determining factor with younger players showing an overall lower level of skating efficiency compared with older players.

Allisse et al. 2017 [33]	18 male 1: 13.1 ± 0.6 y, 50.5 ± 5.9 kg, 161.3 ± 5.8 cm 2: 13.6 ± 0.6 y, 54.2 ± 5.5 kg, 165.3 ± 5.4 cm 3: 14.2 ± 0.6 y, 58.6 ± 6.0 kg, 168.8 ± 4.7 kg elite Bantam from Montreal area in 3 session	30 m sprint forward and backward, 6 × 18 m with and without puck, anaerobic power test	To describe the evolution of morphological, physiological, and skating performance profiles of elite age-group ice hockey players based on repeated measures spread over one season.	Muscular variables improved significantly during and between the two hockey seasons (p < 0.05).

Bond et al. 2018 [34]	40 male, 16.2 ± 0.8 y, 1.76 ± 0.06 cm, 73.7 ± 9.8 kg, top tier Division 1 National Collegiate Athletic Association schools	Top speed, 15.24 m and Multiple repeated sprint test	To determine the reliability of on-ice sprint	All 3 tests may be used in team selection and identification of fatigue or overtraining. The Multiple repeated sprint test may be the most sensitive to short-term improvements related to ice hockey conditioning.

Buckeridge et al. 2015 [14]	9 male, 25.7 ± 3.7 y, 86.2 ± 7.8 kg, 180.0 ± 4.9 cm, 9 r recreational level, 36.9 ± 5.3 y, 86.3 ± 13.8 kg, 177.8 ± 5.6 cm, varsity level	15 × 30 m sprint	In this study a novel on-ice measurement approach was tested for reliability, and subsequently implemented to investigate the forward skating technique, as well as technique differences across skill levels.	The successful implementation of this on-ice mobile measurement approach offers potential for athlete monitoring, biofeedback and training advice.

Bundarick et al. 2020 [15]	9 elite male, 22.0 ± 1.0 y, 1.81 ± 0.08 m, 81.5 ± 8.4 kg, 16 ± 2 exp; 10 elite female, 21.0 ± 1.0 y, 1.72 ± 0.07 m, 71.2 ± 10.4 kg, 14.0 ± 1.0 exp; from Canadian Interuniversity Sport League	Sprint 15 m	To compare body centre of mass and lower body kinematic profiles from static start to maximal speed of high caliber male and female ice hockey players on the ice surface.	Males generated greater forward acceleration during the initial accelerative steps, but thereafter, both sexes had similar stride-by-stride accelerations up to maximal speed. In terms of technique, males demonstrated greater hip abduction (p = 0.006) and knee flexion (p = 0.026) from ice contact to push off.

Bracko and Fellingham 2001 [35]	77 males and 54 females, 19 females 10-11 y (10.95 ± 0.55 y, 143.43 ± 8.30 cm, 36.44 ± 7.13 kg, 3.80 ± 1.74 exp); 21 males 10-11 y (10.75 ± 0.65 y, 140.92 ± 4.11 cm, 35.66 ± 4.32 kg, 5.00 ± 0.89 exp); 20 females 12-13 y (12.75 ± 0.54 y, 158.60 ± 1.07 cm, 46.97 ± 7.95 kg, 4.25 ± 2.27 exp); 31 males 12-13 y (12.25 ± 0.52 y, 159.17 ± 8.68 cm, 48.28 ± 8.54 kg, 6.68 ± 1.06 exp), American hearing Impaired Hockey Association summer camp in Chicago	S-Corner, sprint 6.1 m and 44.80 m	To compare years of playing experience, off-ice fitness, on-ice performance skating, and on-ice anaerobic power of female and male ice hockey players between the ages of 10 and 15 years.	Differences between the female and male hockey players. The 10-11 females accelerated quicker over 6.1 m. In every age group, the males were faster on the speed test. On AGL, the 12-13-year males performed better. In every age group, the males produced more skating Anaerobic power.

Bracko 2001 [36]	23 females, 8 elite: 25 ± 5 y, 176 ± 2.3 cm, 68.9 ± 6.4 kg, 17 ± 6 exp; 15 non-elite: 19 ± 2 y, 168.2 ± 7.6 cm, 65.3 ± 6.6 kg, 8 ± 3 exp	S-Corner test, sprint 6.1 and 47.85 m, 15.20 full speed, Reed repeat sprint skate test	to compare skating performance and on-ice fitness of elite and non-elite women‘s ice hockey players.	These results suggest that elite women‘s ice hockey players are older, faster skaters; have a better drop-off percent, drop-off time, and sum of 6 repeats in a repeat skate test; and have greater on-ice anaerobic capacity than non-elite players.

Bracko and George 2001 [37]	61 females, 8-16 y, 12.8 ± 2.05 y, 155 ± 14.3, 44.4 ± 12.3, 4.68 ± 2.6 exp.	Sprint 6.1 m, 47.85 speed, S-Corner test	To identify the off-ice variables associated with high-performance skating acceleration, speed, agility, and on-ice anaerobic capacity and power in women‘s ice hockey players.	40-yd dash time is the strongest predictor of skatings peed in women‘s hockey players ages 8-16 years old. Prediction equation was speed = 4.913– (0.0107 × kilograms) + (0.4356 × 36 m dash time).

Czeck et. al 2022 [38]	15, males, 20.8 ± 1.4 y, 183 ± 0.04 cm, 85.39 ± 7.55 kg 18 females, 20.86 ± 1.0 y,171 ± 0.4 cm, 74.57 ± 9.02 kg; NCAA Division I collegiate players	9, 18, 24, 42, 48, 66, 82, 132, and 148 m	To explore positional differences for an on-ice timed skate test and its relationship to body composition.	No differences between on-ice skating times between forwards and defensemen. Body fat percentage was correlated with on-ice skate times in male and female collegiate hockey players.

Dæhlin et al. 2017 [39]	16 male, U18 n = 9, 17.2 ± 1.0 y 1.82 ± 0.03 m; U20 n = 7, 17.1 ± 0.7 y, 1.84 ± 0.05 m, NR, elite in Norway;	Sprint 10 and 35 m, skating multistage aerobic test	To compare the effect of combined plyometric and strength training on skating sprint performance	Plyometry and strength training gained a larger improvement in 10 m on-ice sprint performance than strength training (P < 0.025).

Delisle-Houde et al. 2019 [40]	18 male, 22.8 ± 1.4 y, 180.70 ± 6.40 cm, 87.10 ± 6.7 kg, University varsity team	Sprint 30 m forward and backward, On-ice pro agility left and right	To determine the predictability of common laboratory/field and novel laboratory tests for skating characteristics in Canadian college ice hockey players.	Pearson correlations and stepwise regression revealed relationships between on-ice forward sprint and Wingate relative peak power [r = -.62, P < .01], standing long jump [r = -.45, P < .05], off-ice proagility left [r = .51, P < .05], and vertical jump impulse [ r = .60, P < .01].

Haukali and Lief 2016 [41]	18 elite male from Norwegian ice hockey club, T1 (pre test - end of season), 15.8 ± 0.9 y, 68.2 ± 9.6 kg, 176.7 ± 7.7 cm; T2 (start of new season), 16.0 ± 0.7 y, 69.9 ± 8.7 kg, 177.0 ± 7.6 cm; T3 (midseason), 16.2 ± 0.6 y, 70.7 ± 9.0 kg, 178.0 ± 7.7 cm	Sprint 36 m forward and backward, The Norwegian Hockey Federation s skate agility test.	To examine whether there exists a correlation between changes in off-season power and changes in in-season skating performance among young ice hockey players	An off-season training program that includes sprints and horizontal jumping exercises may have a positive effect on hockey players’ linear skating speed.

Haukali and Lief 2015 [42]	32 juniors male, 16.4 ± 0.6 y, 70.9 ± 5.2 kg, 179.3 ± 5.2 cm	Sprint 36 m, skating speed 36 m, The Norwegian Hockey Federation‘s skate agility test.	To examine the relationship between specific off-ice variables and skating speed and agility among young ice hockey players.	No significant correlation between agility and speed skating, or between agility and sprint. Off-ice training program that includes sprint training and jumping exercises will have a positive effect on young hockey players’ skating performance.

Farlinger and Fowles 2008 [3, 43]	34 juniors male and female, 16.4 ± 2.0 y, 70.4 ± 12.8 kg, 174.5 ± 4.2 cm	Sprint 35 m	To determine the effectiveness of a progressively “skating specific” periodized off-season training program on skating performance in competitive hockey players.	Significant improvements in on-ice 35-m skating sprint (1.0%; P = .009) with significant improvements of 5% to 12% in various off-ice testing measures were observed. On-ice 35-m skating sprint times improved by 2.3% with greater improvement in plyometric followed by simulator training (3.5%) versus simulator training followed by plyometric training (0.8%).

Farlinger et al. 2007 [3]	36 males between 15–22 y, 16.3 ± 1.7 y, 175.6 ± 4.1 cm, 70.8 ± 10.4 kg,	Sprint 35 m, S-Corner test	To identily off-ice variables that would correlate to on-ice skating sprint performance and cornering ability.	The on-ice sprint test and cornering S test were strongly correlated (r = 0.70; p < 0.001). While many office tests correlated with on-ice skating, measures of horizontal leg power (off-ice sprint and 3 hop jump) were the best predictors of on-ice skating performance, once weight and playing level were accounted for. These 4 variables accounted for a total of 78% (p < 0.0001) of the variance in on-ice sprint performance.

Federolf and Nigg 2008 [20]	20 males, 22.6 ± 0.9 y, 184 ± 5 cm, 87.3 ± 7.3 kg from University of Calgary ice team, NR	Maximum speed 45 m, Glide turn test, Sprint 10 m and 40 m	To test if a flared blade design measurably improves skating performance of ice hockey players in selected skating tests.	The results of this study show that relative simple modifications on ice hockey equipment can lead to measurable improvements in skating performance.

Geithner et al. 2006 [44]	112 female players, 21.4 ± 2.9 y, 168 ± 5 cm, 66 ± 7 kg, University of Alberta, Hockey Team the Pandas	Sprint 6.1 m, S-Corner Test, Modified 3-Repeat Sprint Skate Test	To describe and compare physical, fitness, and skating performance characteristics of forwards, defensemen and goalies.	Forwards had greater anaerobic power than defense, followed by goalie, and they tended to have greater aerobic capacity. Performance demands appear to the position specific.

Gilenstam et al. 2011 [13]	10 male, 23.0 ± 2.4 y, 181 ± 5 cm, 85.0 ± 7.8 kg; 11 females, 23.0 ± 3.0 y, 168 ± 3.5 cm, from 2^nd^ division in Sweden	Sprint 6.1 and 47.85 m, S-Corner test, Full speed test for 15.2 m	To identify relationships between physiological off-ice tests and on-ice performance in female and male ice hockey players on a comparable competitive level.	Skating performance in female hockey players may be improved by increasing thigh muscle strength, oxygen uptake, and relative muscle mass.

Gupta et al. 2022 [45]	19 male juniors; 18.7 ± 2.2 y, 179.2 ± 11.6 cm, 77.8 ± 10.2 kg, from club UKS Zagłebie Sosnowiec, Poland	Skating Multistage Aerobic Test, sprint 5-10-30 m, Top speed test, Repeated sprint ability 5 × (54 + 35 m), Vertical jumps	To determine the relationships between vertical jumps and various on-ice skating performances of junior ice hockey players	Depth drop jump can be used as a predictor of all the ice skating speed tests, whereas squat jump can predict backward average skating speed and maximum skating speed. Repeated sprint ability performance cannot be predicted from vertical jumps of junior ice hockey players.

Hajek et al. 2021 [46]	10 male, U18 = 16.0 ± 1.0 y, 1.75 ± 0.1 m, 68.0 ± 9 kg; U14 = 12.0 ± 1.0 y, 1.57 ± 0.08 cm, 49.0 ± 9 kg; U18 and U14 top Austrian National Team	Sprint 40 m	To develop and validate a specific overall skating performance test for ice hockey (SOSPT)	The results of the study reveal reliability and validity of specific overall skating performance in ice hockey players and is more suitable compared with straight skating tests of the 40-m on-ice sprinting test.

Janot et al. 2015 [47]	26 male and females, 20.5 ± 1.4 y, 1.74 ± 1.0 m, 79.6 ± 13.5 kg, 14.0 ± 4.3 exp, NCAA Division III	S-Corner test, sprint 6.1, 15.20 and 44.8 m, Modified Repeated Skate	To determine if off-ice performance variables could predict on-ice skating performance	It was concluded that selected off-ice tests could be used to predict on-ice performance regarding speed and recovery ability in Division III male and female hockey players.

Janot et al. 2013 [48]	23 male players, 12.3 ± 1.1 y, 51.7 ± 18.5 kg, NR	Sprint 6.1, 15.2 and 44.8 m	Determine if on-ice BungeeSkate traing would improve on-ice speed and acceleration	Speed and top speed were significantly increased (P < .05) by 4.2% and 4.3%, respectively.

Jones et al. 1999 [49]	16 male, 27 ± 4 y, 1.81 ± 0.07 m, 85.5 ± 8.2 kg, British Super League Club	6 × 80 m skating sprint every 30 s, split taken at 47 m	To assess the effect of oral creatine monohydrate supplementation on multiple sprint cycle and skating performance in ice-hockey players.	Average on-ice sprint performance on 47 m was significantly faster at 10 days 6.88 ± 0.21 sec) a d 10 weeks 6.96 ± 0.19 sec than at baseline 7.17 ± 0.27 sec (p < 0.005).

Kaartinen et al. 2021 [50]	12 elite males, 18.4-22.0 y, 81.0 ± 6.0 kg, 1.82 ± 0.03 m, 25.2 ± 1.6 BMI kg/m^2^, Finnish elite league	5 × 30 m sprint	To describe lower limb kinematic and muscle activation patterns and then to examine the potential associations between those variables and skating speed	A lower activity of the gluteus maximus (r = -0.651, p = 0.022, β = -0.08) and a reduced gluteus maximus to rectus femoris coactivity (r = -0.786, p = 0.002, β = -3.26) during the recovery phase were found to be associated with faster skating speed.

Kinnunen et al. 2019 [51]	14 females, 22 ± 3 y, 16-28 y 165 ± 5 cm, 67 ± 12 kg, Finnish championship	Sprint 11 and 34 m	To investigate High-intensity training related to neuromuscular adaptations, changes in force production, and on-ice performance in female ice hockey players during preseason.	High-intensity training can be used to improve athletes' capability to produce maximal and explosive forces, likely through enhanced voluntary activation of their muscles and reduced antagonist coactivation.

Lagrange et al. 2020 [52]	41 elite male, Group n = 21, 16.8 ± 1.3 y, 177.3 ± 6.1 cm, 166.8 ± 8.6 lbs, 24.1 ± 2.0 BMI,; Control group n = 20, 16.4 ± 1.7 y, 181.6 ± 5.3 cm, 169.6 ± 7.3 lbs, 23.6 ± 2.1 BMI, midget or junior hockey players	9 sprints of 40 m each (approximately 5 seconds) with 3 s rest	To measure the generating effects of Contrast Training on 6-hour post-activation potentiation and its influence on jumping and on on-ice repeated sprint performance in ice hockey players.	The contrast training generated PAP which had an acute performance enhancement on the on-ice hockey repeated sprint test performance.

Martini et al. 2018 [53]	59 male, 14.6 ± 2.1 y, 166.4 ± 12.8 cm, 60.4 ± 14.1 kg, 21.5 ± 2.6 BMI, 8.9 ± 3.1 exp.	Sprint 6.1 and 44.8, 7 × 7 m	To measure ice hockey players skills and analyze their fluctuations via a protocol that reproduces the demands of a hockey game	A significant decline in performance was observed for speed, acceleration, and shooting (p < .01). Inversely, participants seemed to adapt to puck control and passing stations, as they became faster without decreasing skating abilities.

Madden et al. 2019 [54]	15 college males (SP: skill performance), 22.1 ± 0.4 y, 83.3 ± 1.8 kg, 182 ± 2 cm; and 14 college males (scrimmage), 22.6 ± 0.4 y, 85.8 ± 2.3 kg, 182 ± 2 cm; both from Okananan Hockey Group and completed Western Hockey League Combines	Sprint 30 m forward and backward, transition agility, weawe agility, reaction with and without puck	To determine the effects of low-dose caffeine supplementation (3 mg/kg body mass) consumed 1 h before the experiment on rating of perceived exertion, skills performance (SP), and physicality in male college ice hockey players	A low dose of caffeine has limited impact on sport-specific skill performance and rating of perceived exertion but may enhance physicality during ice hockey scrimmage.

Naimo et al. 2015 [55]	24 male a, interval group: n = 12, 19.6 ± 1.3 y, 180.0 ± 7.0 cm, 79.9 ± 8.3 kg; continuous group: n = 12, 19.7 ± 2.1 y, 174.9 ± 7.9 cm, 74.9 ± 9.0 kg, Universtiy of Tampa	6 × 9 m stops, sprint 33 m, 127 m endurance test	To investigate the efects of a High intensity interval training program compared to traditional continuous endurance exercise training	High intensity interval training demonstrated a faster Δ sprint (p < 0.02) and a trend (p = 0.08) for faster Δ endurance test time to completion for IG.

Matthews et al. 2010 [56]	11 male, 22.09 ± 3.05 y, 83.47 ± 11.7 kg, 179 ± 6 cm, from the English National	Sprint 25 m	To investigate the acute effect of a heavy resisted sprint when used as a preload exercise to enhance subsequent 25-m on-ice sprint performance	These findings appear to suggest that the intensity and duration of a single resisted sprint in this study are sufficient to induce an acute (after 4 minutes of rest) improvement in 25-m sprint performance on ice.

Nigg 2020 [32]	20 male 1 female: 36.25 ± 12.91 y, 178.85 ± 8.64 cm, 81.36 ± 12.89 kg, 5.68 ± 8.93 exp, 5^th^ German Player League,	S-Corner test, sprint 6.1 m and 35 m	Investigate which performance indicators were related to the plus-minus statistic in German recreational ice hockey players over a season.	Some performance and psychological indicators are related to recreational ice hockey players’ plus-minus statistic over a season.

Novak et al. 2019 [8]	14 males: 14.8 ± 0.45 y, 61 ± 10.43 kg, 168.93 ± 9.72 cm, 9.07 ± 0.75 exp, highest junior league in Czech republic	Sprint 6.1 and 35 m, S-Corner test, test with brake, weave agility, reaction test	To compare the effects of on-ice and off-ice agility training on skating performance.	Off-ice agility have motor transfer to on-ice agility. Therefore, we recommend developing on-ice agility with additional off-ice agility training during the ice hockey season.

Novak et al. 2020 [57]	13 males from U12 in Czech republic, 13 ± 0.35 y, 152.23 ± 9.41 cm, 41.92 ± 8.76 kg, 17.91 ± 2.14 BMI	7 × 7 with and without puck, sprint 30 m forward and backward, 4 m sprint forward and backward with and without puck.	to compare the effects of training programs using change-of-direction speed exercises and partial skating task training on speed and agility performance in U12 ice hockey players.	Change of directional speed training on the ice improves short starts and agility with a puck, while partial skating tasks training target longer 30-m sprints and agility without a puck.

Peterson et al. 2015 [58, 59]	45 male between 18-24 from, Division I (n = 24): 184.92 ± 5.99 cm, 86.88 ± 6.69 kg, 11.46 ± 2.43 %fat; Elite Junior (n = 10): 180.70 ± 6.78 cm, 82.30 ± 5.62 kg, 9.40 ± 2.01 %fat; Division III (n = 11): 178.55 ± 3.50 cm, 82.30 ± 4.95 kg, 14.36 ± 2.29 %fat, Division I and III and Elite Junior level in Minneapolis	Repeated shift test	To examine the differences that may exist between these characteristics in Division I, Elite Junior, and Division III hockey players	No significant difference between Division I and Elite Junior players for any on or office performance variable. The results of this study indicate that performance differences between Division I and Division III hockey players seem to be primarily because of the rate of force production.

Peterson et al. 2016 [59]	45 male 18-24 y, 181 ± 9 cm, 84 ± 12 kg, 12.5 ± 4 %fat, Division I, II or Junior Level hockey in Minneapolis	Sprint 15.24 m, Repeated shift ability	To test whether conventional off-ice anaerobic power tests could predict on-ice acceleration, top speed, and repeated shift performance	Although conventional off-ice anaerobic power tests predict single bout on-ice acceleration and top speed, they neither predict the repeated shift ability of the player, nor are good markers for performance in ice hockey.

Perez et al. 2021 [17]	17 female, 21.6 ± 3.4 y, 166 ± 9 cm, 65.3 ± 9.9 kg, 14.6 ± 3.2 exp, elite French national team,	skating (5 and 40 m) sprint tasks	To investigate the correlations between players’ mechanical capacities determined during off and on-ice tests	Performance variables (SJ height, 30-m running and 40-m forward skating split time) and Pmaxrel demonstrated the largest associations between jumping, running and skating tasks (r ranging from -0.81 for 30-m sprint running time to 0.92 for SJ height; p < 0.001). The capacity to generate high amounts of horizontal power and effective horizontal force during the first steps on the ice is paramount for forward skating sprint performance.

Perez et al. 2022 [60]	11 highly-trained females, 17.6 ± 4.0 y, 1.66 ± 0.06 m, 63.6 ± 6.3 kg, 10.5 ± 4.1 exp, French	Sprint 2 × 40 m	To ensure that the skating velocity describes a mono-exponential function in order to determine the reliability of radar-derived profiling results from skating sprint accelerations applying sprint running force-velocity assessment approach.	The current study indicates that radar-derived kinetics variables assessed during onice 40-m forward sprint skating demonstrate an acceptable level of relative and absolute reliability.

Renaud et. al. 2017 [10]	15 males, 7 high calibre males, 24.7 ± 3.1 y, 184.2 ± 6.4 cm, 87.1 ± 6.0 kg, 19.7 ± 3.9 exp; 8 low calibre males, 23.9 ± 3.1 y, 179.4 ± 3.4 cm, 81.3 ± 8.4 kg, 9 ± 6 exp, Montreal, CA.	3 repetition of a maximum effort forward skating start	To evaluate kinematic ice hockey skating start movement technique in relation to a skater's skill level.	Both skate groups had similar lower body strength profiles, yet high calibre skaters achieved greater velocity; skating technique differences most likely explained the performance differences between the groups.

Robert-Lachaine et al. 2012 [27]	10 males, 22.9 ± 2.2 y, 80.5 ± 9 kg, 178.3 ± 4.5 cm, 16.8 ± 3.4 exp	Crossover on outside leg (COO) and inside leg (COI), forward skating	To determine if these modifications resulted in biomechanical and performance changes during on-ice skating skills.	Modified skates demonstrated significant gains of 5°-9° in dorsi-plantarflexion ROM (p < 0.05). Total peak force occurred later during plantarflexion, suggesting a more prolonged and effective force generation with the modified skates during a given skating stride.

Roczniok et al. 2016 [61]	42 males, G1: 20, 24.70 ± 3.53 y, 182.9 ± 4.02 cm, 78.48 ± 7.15 kg, 24.19 ± 1.52 BMI; G2: 22, 24.82 ± 6.68 y, 179.18 ± 5.22 cm, 80.20 ± 9.27 kg, 24.94 ± 2.39 BMI, Poland top division	Sprint 30 forward and backward, 6 × 9 turns, 6 × 9 stops, 6 × 30 stops	To examine physiological and physical determinants of ice-hockey performance	The logistic regression model showed that the best predictors of success in the recruitment process of top level ice hockey players were time to peak power, relative peak power, VO_2max_ and 30 m sprint forwards on ice.

Roczniok et al. 2016 [62]	24 elite males, season 2012/2013 = 177.70 ± 4.19 cm, 79.43 ± 8.28 kg; season 2013/2014 = 177.80 ± 4.37 cm, 80.62 ± 8.19 kg, NR, Poland second league	Sprint 30 forward and backward, 6 × 9 turns, 6 × 9 stops, 6 × 30 stops	To determine the values of selected aerobic and anaerobic capacity variables, physical profiles, and to analyze the results of on-ice tests performed by ice-hockey players relegated to a lower league.	The study showed that playing in a lower league where games were less intensive, training sessions shorter and less frequent, had an adverse effect on the performance level of the investigated players.

Roczniok et al. 2012 [63]	21 males, NR, NR, Polish National Team, U20 World Championship 2009	Sprint 30 forward and backward, 6 × 9 turns, 6 × 9 stops, 6 × 30 stops	To determine the predictive value of the indexes of aerobic and anaerobic endurance in relation to specific on-ice tests performed by hockey players that focus on strength, power, speed as well as speed and strength endurance.	The obtained results found significant correlations between maximal power obtained from the Wingate test and certain aspects of the special physical fitness test, specifically the 6 × 9 turns, 6 × 9 stops and 6 × 30 m endurance tests. Significant correlations of the above-mentioned special physical fitness tests were also observed with the aerobic capacity parameter, VO_2max_.

Rønnestad [64] et al. 2016 [64]	15 males from 2^nd^ level in Norway, 21 ± 2 y, 79 ± 7 kg, 180 ± 5 cm	Sprint 10 and 20 m	To investigate the effect of body-loaded half-squats with added whole body vibration on subsequent 20 m on-ice sprint performance.	Improved well-being in the legs immediately after the preconditioning exercise with whole-body vibration (p < 0.05). Preconditioning exercise performed with WBV at 50 Hz seems to enhance on-ice sprint performance in ice-hockey players.

Runner et. al. 2016 [65]	40, elite male, NR, 181.30 ± 5.80 cm, 84.95 ± 5.58 kg, National Collegiate Athletic association Division I	27.4 m forward and backward acceleration, 15.2 m flying top speed test	To examine the relationship between commonly employed dry-land performance tests and skating speed in male collegiate ice hockey players.	Vertical jump showed significance in relation to skating speed (p = 0.011).

Shell et al. 2017 [66]	10 elite females, 21 ± 1 y, 14 ± 1 exp, 1.72 ± 0.07 m, 71.2 ± 10.4 kg; 9 elite males, 22 ± 1 y, 16 ± 2 exp, 1.81 ± 0.08 m, 81.5 ± 8.4 kg, players from Canadian Interuniversity sport league	Sprint 15.3 m	To evaluate the body movement kinematics of ice hockey skating starts between elite male and female ice hockey participants	Males' maximum skating speeds were greater than females. Females presented ~10° lower hip abduction throughout skating stance as well as ~10° greater knee extension at initial ice stance contact, conspicuously followed by a brief cessation in knee extension at the moment of ice contact, not evident in male skaters.

Secomb et al. 2021 [9]	13 male, 26.7 ± 6.7 years; 88.4 ± 18.1 kg; 181.9 ± 5.4 cm), semiprofessional males from Australian Ice Hockey League	Sprint 20 m, On-Ice Change of Direction (505)	To determine whether hip strength in joint angles specific to skating positions and countermovement jump performance explains sprint skating acceleration and change of direction performance.	Hip strength at joint angles functionally relevant to skating (e.g., at 25° and 50°), in combination with relevant countermovement jump variables, explained large and very large amounts of variance in sprint skating acceleration and change of direction performance in this cohort.

Schulze et al. 2021 [67]	104 male elite, 26.4 ± 5.62 y, 1.82 ± 0.06 m, 86.9 ± 8.70 kg, 64 forwards, 40 defenders, professional German Forward 3^rd^ league	Ice-hockey specific complex test (sprint 10 and 30 m, transition and weave agility with and without puck, slap and wrist shot, stress)	To investigate position-specific reference data for Ice-hockey specific complex test	The only significant (p < 0.002) difference between forwards and defenders for performance were found for weave agility with puck (p < 0.001). Forwards showed a higher average performance in this parameter than defenders. Differences were also found in weave agility without a puck (p = 0.008), 30 m backward sprinting without puck (p = 0.012) and goals after test (p = 0.030).

Schwesig et al. 2018 [68]	20 male elite from Third German League, 27.0 ± 5.8 y, 1.82 ± 0.05 m, 88.3 ± 7.7 kg	10-m sprint, a 30-m transition with and without a puck	This study assessed the intra-rater reliability of an ice hockey-specific complex test that reflects the intense multidirectional and intermittent efforts required in ice hockey	The Ice-hockey specific complex test is currently the only ice hockey-specific complex test with scientifically tested reliability and validity that can analyze performance under conditions similar to the competition.

Schwesig et al. 2017 [69]	18 elite male, Third Professional German League, age 27 ± 6.0 y, 1.82 ± 0.05 m, 89.8 ± 7.5 kg	Ice-hockey specific complex test (sprint 10 and 30 m, transition and weave agility with and without puck	To examine the validity of an ice hockey-specific complex test Ice-hockey specific complex test and nonspecific off-ice tests for sports performance.	Stability indicator (r^2^ = 0.39), weave agility with puck (r^2^ = 0.39), maximal relative squat (r^2^ = 0.37) and frequency band -8 (r^2^ = 0.35) proved to be the most valid tests. However, with the match performance score as the dependent variable, 21 of 44 parameters tested (48%) explained 10% or more of variance.

Stenroth et al. 2020 [71]	12 male, 18.4-22.0 y, 83.9 ± 6.2 kg, 1.82 ± 0.03 m, 25.2 ± 1.8 BMI, eliteFinnish junior league	Sprint 30 m	The reliability and concurrent validity of force-velocity profiling based on split times when used for ice hockey skating. Which the start instant of the sprint is estimated based on optimization (time shift method), affects the reliability and validity of the method.	The time shift method tested here can be used as a reliable tool to test a player's physical performance characteristic underlying sprint performance in ice hockey skating.

Stetter et al. 2019 [72]	22 male, 32.1 ± 7.7 y, 178.7 ± 5.7 cm, 86.7 ± 10.5 kg, Calgary men's hockey team	Max effort 30 m sprint	To investigate the feasibility of using body worn accelerometers to identify previous highlighted performance-related biomechanical changes in terms of substantial differences across skill levels and skating phases.	High caliber players showed an increased stride propulsion (+22%, P < 0.05) and shorter contact time (-5%, P < 0.05). All three analysed variables highlighted substantial biomechanical differences between the accelerative and constant velocity phases (P < 0.05). Stride propulsion of acceleration strides primarily correlated to total sprint time (r = -0.57, P < 0.05).

Thompson et al. 2020 [73]	42 male, 23.0 ± 2.07 y, 175 ± 10 cm, 77.3 ± 12.5 kg, 24.98 ± 2.16 BMI University of Guelph	Sprint 15 m	To compare the suitability of common off-ice fitness tests and off-ice resisted sprints for predicting 15 m on ice skate time.	We conclude that resisted off-ice sprints have better predictive ability of on-ice skate time compared with commonly used off-ice tests.

Stanula et al. 2014 [74]	24 male, 25.2 ± 3.93 y, 182.9 ± 3.7 m, 86.9 ± 6.1 kg National Hockey Team of Poland,	Repeated Skate Sprint (6 × 89) with 30 rest	To determine a relationship between aerobic capacity (VO_2max_) and fatigue from high-intensity skating	The 6 × 89 m test proposed in this study offers a high test-retest correlation coefficient (r = 0.78).

Stanula et al. 2021 [75]	19 male, forwards = 12, 23.4 ± 4.76 y, 179.8 ± 5.68 cm, 80.5 ± 7.57 kg; defenders = 7, 22.3 ± 5.2 y, 182.0 ± 3.46 cm, 87.1 ± 4.81 kg, Zagłebie Sosnowiec, Poland	Repeated Skate Sprint	The impact of two different passive recovery durations, two and three minutes, between sets of repeated sprint skating ability test on skating speed, speed decrement, and heart rate response.	This study concludes that three-minute recovery is beneficial over two-minute recovery by increasing skating speed.

Vigh-Larsen et al. 2020 [6]	30 male, 19.0 ± 1.0 y, 184.3 ± 6.2 cm, 83.4 ± 8.8 kg, 42.9 ± 5.2 muscle mass, 10.3 ± 0.7 %fat, Danish U20 national team	Repeated sprint ability	The present study investigated muscle metabolism and fatigue during simulated elite male ice hockey match-play.	Repeated-sprint ability was impaired (—3%; P < 0.05) postgame concomitant with a —10% decrease in the number of accelerations and decelerations during the second and last periods (P < 0.05).

Vigh-Larsen et al. 2020 [4]	Male 179, Finish elite: 74, 25.9 ± 5.4 y, 183.0 ± 6.4 cm, 86.1 ± 7.8 kg, Danish Elite: 55, 23.9 ± 4.5 y, 182.3 ± 6.0 cm, 84.3 ± 8.3 kg, Finish U20: 19, 18.7 ± 0.9 y, 180.0 ± 7.2 cm, 78.6 ± 8.4 Danish U20: 17, 18.1 ± 1.7 y, 79.0 ± 5.9 cm, 75.7 ± 10.1 kg,	5-10-5 Pro-agility, sprint 10 and 30 m, Yo-Yo Intermittent Recovery Level 1 Ice Hockey Test	To compare fitness profiles and body composition in elite players of 2 different national standards in a large sample of players, applying specific on-ice and off-ice test procedures. To compare the fitness level of players of these elite standards with U20 players from both countries to test for potential age-related differences.	Large differences in on-ice performances were demonstrated between Finnish and Danish elite players for agility, 10- and 30-m sprint performance. Finnish U20 cohort had a similar performance level as the Danish elite players and superior 10-m sprint performance.

Vigh-Larsen et al. 2019 [5]	164 elite male 23.5 ± 4.4 y, 182.3 ± 5.2 cm, 85.7 ± 8.1 kg, 15.1 ± 4.0 %fat; 132 subelite 19.4 ± 2.9 y, 180.9 ± 6.8 cm, 80.8 ± 10.0 kg, 15.6 ± 6.5 %fat, Best and second Danish ice hockey division.	Yo-Yo heart rate max and submax, 5-10-5 proagility test, 0-10.85 and 10-33.15 sprint	To evaluate fitness profiles in elite (and subelite male ice hockey players.	In conclusion, elite-level ice hockey requires a high level of fitness in terms of muscle mass and explosive strength, as well as a well-developed high-intensity intermittent exercise capacity. In addition, these demands seem to apply for both forwards and defensemen.

Vigh-Larsen et. al. 2021. [76]	199 males: 65, 18-21 y, 182.5 ± 5.9 cm, 83.0 ± 8.2 kg, 71, 22-25 y, 183.5 ± 5.5 cm, 87.1 ± 7.2 kg, 40, 26-29 y, 181.5 ± 5.1 cm, 86.4 ± 6.4 kg, 23, 30-33 y, 184.2 ± 5.4 cm, 91.2 ± 9.6 kg	5-10-5 Pro Agility test, sprint 30 m, Yo-Yo heart rate max	To investigate relationships between age, body composition and performance in elite male ice hockey players.	Small-moderate associations between age and body composition were present. Lower high-intensity exercise performance was evident in the oldest and a lower body weight in the youngest players, whereas aerobic capacity was similar. This suggests that capabilities related to size, strength and power are the most critical parameters differing between young and old ice hockey players.

Wagner et al. 2021 [77]	13 = U15, 13.3 ± 0.5 y, 1.62 ± 0.1 m, 56 ± 10 kg; 18 = U17, 15.1 ± 0.7 y, 1.76 ± 0.7 m, 68.0 ± 9.0 kg; 19 = U20, 17.8 ± 2.0 y, 1.78 ± 0.05 m, 76.0 ± 5.0 kg; Swiss elite league	Sprint 6 and 30 m, specific overall skating performance test	To analyze the differences in on-ice and off-ice performance and relation between on-ice and off-ice performance	The stronger relationship between specific overall skating performance test performance and on-ice and off-ice performance in the younger compared to the older players revealed that general physical performance determined specific overall skating performance more often in youth players, whereas in junior und young adult players, an optimal skating technique is more important.

Williams and Grau 2020 [78]	12 male, 17.92 ± 0.9 y, 185 ± 8.45 cm, 83.17 ± 8.61 kg elite adolescent	Sprint 15 m, S-Corner test	To analyse the relationships between physical performance and game performance, as well as examining the relationships of physical performance and game performance dependent on competition level.	Results illustrated an inverse relationship between plyometric performance and offensive point shares. On-ice agility may be a relevant indicator of game performance dependent on competition level for individuals and sub-groups.

Wilson et. al 2021 [79]	17 females, 21 ± 2 y, 166.2 ± 6.4 cm, 61.9 ± 7.7 kg	7 × 15 m sprints everç 15 s	To design and examine the reliability of a 7 × 15 m repeated on-ice skating sprint test for female ice hockey players	Players in the forward position had a faster mean 15 m time and lower total time compared to those in the defensive position (p < 0.05). These findings show that a 7 × 15 m repeated on-ice sprint test for varsity women ice hockey players was reliable. It was also found that forwards had a better mean of 7 sprint time and faster total time compared to players in the defensive position.

The full text screening resulted in excluded 41 studies which did not report the results of a straight sprint test. One study was excluded [80] due to reporting in unstandardized values. All other studies met the quality standards. The acceleration data from one study [15] were excluded as they repeated values from previously published data [11], however this study included additional information for a 34 m sprint which was included.

**FIG. 2:**
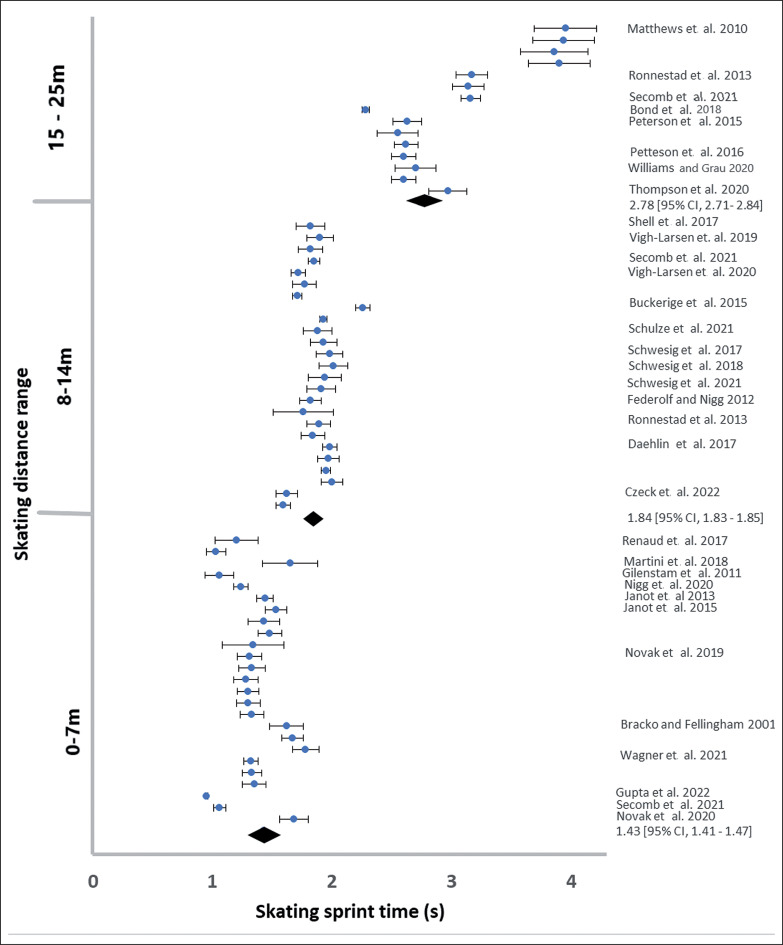
The reported male sprint times during acceleration up to 25 m.

**FIG. 3:**
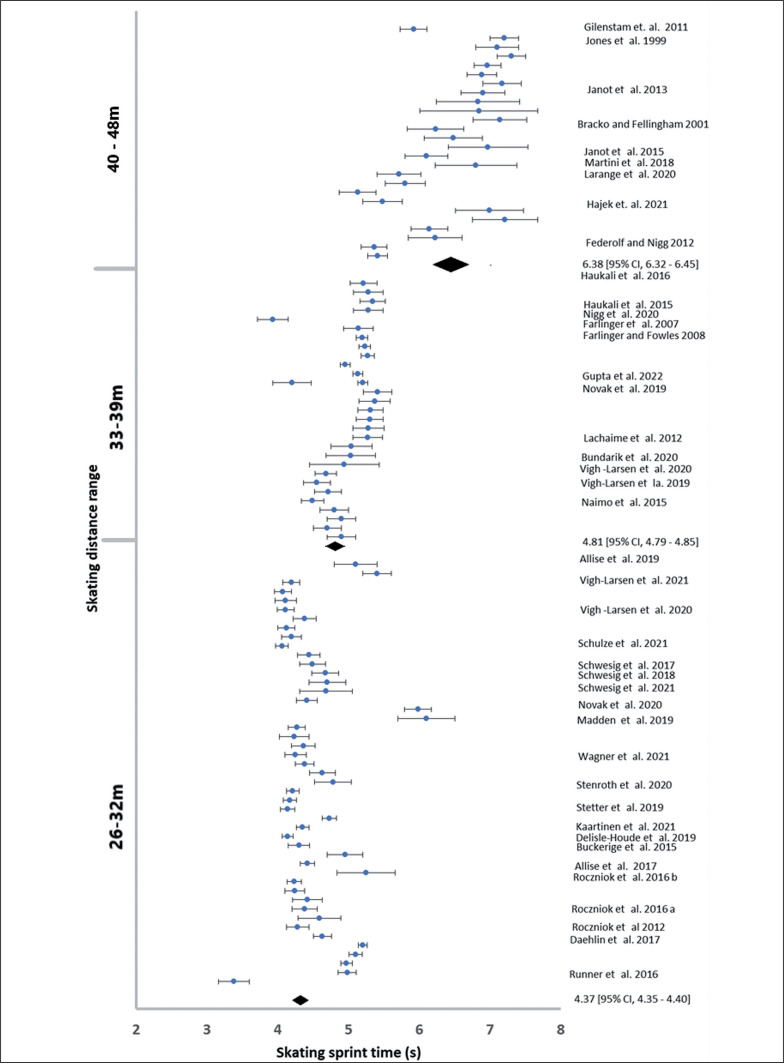
The reported male sprint times during tests between 26 and 48 m distance.

**FIG. 4:**
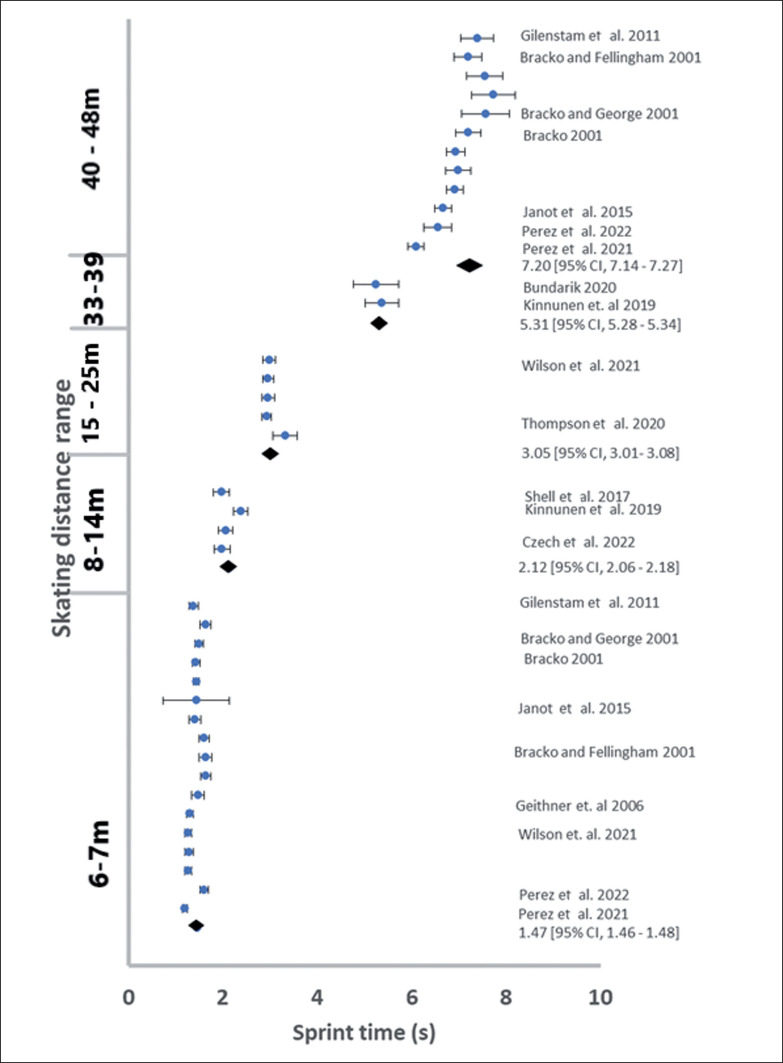
The reported female sprint times during skating sprint tests.

After data extraction there was a pooled sample of 2254 men and 398 women, where each participant is only counted once even if two or more tests (e.g., 6.1 and 30 m) were reported. The pooled data for men had a minimum of 201 participants and 14 studies in each distance range (Table 2; Figures 2 and 3). The pooled data for women was not large enough to permit statistical analysis (Table 2, Figure 4).

**TABLE 2:** Basic data of mean acceleration and speed pooled for analysis.

Distance range (m)	0–7	8–14	15–25	26–32	33–39	40–48
**Men**						
Number of mean values included	25	25	15	48	29	25
Number of subjects	325	663	201	928	445	246
Acceleration (m/s^-2^) Mean ± SD)	3.43 ± 1.00*	2.92 ± 0.45*	2.03 ± 0.37*	1.51 ± 0.29††	1.38 ± 0.20††	1.07 ± 0.90**
Speed (m/s, Mean ± SD)	4.45 ± 0.75*	5.43 ± 0.53*	6.07 ± 0.42*	6.68 ± 0.63†	6.89 ± 0.44†	6.78 ± 0.57†

**Women**						
Number of mean values included	17	4	5	0	2	12
Number of subjects	317	42	39	0	24	160
Acceleration (m/s^-2^) Mean ± SD)	2.80 ± 0.40	2.45 ± 0.59	1.64 ± 0.14	-	1.21 ± 0.03	0.92 ± 0.09
Speed (m/s, Mean ± SD)	3.98 ± 0.30	5.09 ± 1.01	4.96 ± 0.22	-	6.41 ± 0.08	6.43 ± 0.39

* significantly different to all other distance ranges,

† higher than 0–7 m, 8–14 and 15–25 m distance range.

†† lower than 0–7 m, 8–14 and 15–25 m distance and similar to other distances,

** lower that all other distance ranges.

**FIG. 5:**
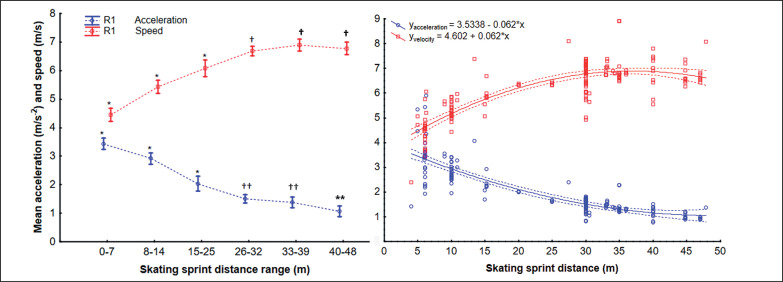
Differences of forward skating acceleration and speed by testing distance (left graph) and their regression fit equation (right graph) with 95% confidence interval.

The assumption of data normality and equality of variance was violated for men in the distance range 40–48 m. The ANOVA analyses showed differences between mean acceleration Welch F_5, 164_ = 66.08, p < 0.001 and speed F_5, 164_ = 71.9, p < 0.001 at different sprint distance ranges. The skating acceleration increased between the 0–7 m, 9–14 m, 15–25 m and 26–32 m distance ranges, and did not differ between 26–32 m, 33–39 m (Figure 5). The 40–48 distance showed lower acceleration than all other distance ranges (Figure 2). The skating speed increased between the 0–7 m, 9–14 m, 15–25 m and 26–32 m distance ranges, and did not differ between 26–32 m, 33–39 m and 40–48 m (Figure 5, Left).

The correlation analyses showed an inverse relationship between straight sprint distance and mean acceleration (τ = -0.73, R^2^ = -0.71, p < 0.001) and a positive relationship between test distance and speed (τ = 0.52, R^2^ = 0.49, p < 0.001) (Figure 5, Right).

## DISCUSSION

As hypothesize the forward sprint maximum acceleration was found in distances up to 7 m, on the other hand the presumption for maximum sprint speed was different since maximum velocity have been found between 26 and 39 m (Figure 5). The reported acceleration for the shortest distance range (0–7 m) was 3.31 m/s^2^, which represents the four intensive skating strides [10] needed during 10% of the game time to compete for the puck or position [70, 81]. Moreover, this short acceleration is necessary for any longer sprint and before [82] or after change of direction maneuvers. Considering that seven of the eleven previous studies used an acceleration distance of 6.1 m (Table 1), this distance should be used for future acceleration evaluation to be easily comparable with to date findings. In the speed evaluation the distances between 25 m and 39 m resulted in a similar average speed (6.68–6.89 m/s), where 30 m was reported most frequently (in 23 studies, Table 1). The 25–39 m distance typically means the transition between the defensive and offensive zones, where the players’ actions include puck carry, and the dump in and out the offensive and defensive zones [83]. Although there is no speed difference in the distance ranges, there is an expectation that the most frequently used 30 m distance is the foundation for defensive to offensive transition and provides the same speed information as longer sprints. Therefore, we recommend using 30 m distance as a standard for future research.

The regression function (Figure 5) showed that ice-hockey sprint speed increases along with skating distance (τ = 0.52) up to 39 m but then plateaus or possibly decreases over longer distances. Moreover, the reported values over 40 m showed more variability and the longer track might not differentiate whether players developed near maximum velocity quickly enough. This plateau in skating speed for longer than 39 m, might be also caused by the limit of the ice-hockey ring (61 m) when sprinting over 40 m the finish will be uncomfortably close to the rink barrier (since the sprint skating instructions should be to skate to maximum to 45 m target). At the same time as the average speed increases, the average acceleration decreases (Figure 5), which is specifically evident for short distances during each skating stride [15]. Therefore there is a need to also evaluate the skating technique at which the sprint performance was done as players have to be skilled in the propulsion, swing phase and glide typical for larger stride lengths and wide in highly trained players [24]. Therefore, a critical metric for skating technique is the stride count for the distance covered, which is included e.g. in skating efficiency index for sprints (between stride and time r = 0.342) [28], and might be evaluated automatically from a single accelerometer on skate [84]. Thus the ices-hockey sprint testing protocol for any distance should include the stride count along with reported time, acceleration, and velocity.

One of the crucial aspects of conditioning testing is the game requirements and performance relevance. Although sprint performance has been clearly associated with ice-hockey performance levels [5, 85, 86], the evidences in skating abilities during real games are scarce [23]. Moreover, the current method of tracking players’ acceleration and velocity within a match is based on local positioning systems (LPS) or accelerometers, which do not allow direct transfer to testing by photocells out of match conditions. For example, we found the average speed of longer maximum effort sprints (26–48 m) to be 24.3 ± 2 m/s, but match analyses differentiate between skating intensities (fast speed and sprint) using a value of 24 km/h [21, 22]. This difference might be caused by the speed/velocity calculation as when the straight sprint is measured by photocells only the average speed in one direction can be calculated, whereas LPS and accelerometry systems measure the velocity in any direction including side to side direction of stride during forward skating, crossovers, and other changes of direction. However, the velocity ranges [21, 22] currently used in match analyses are taken from soccer recommended values, where the motion does not include any gliding. Therefore, the sprint velocity borderlines for LPS systems should be validated in a separate study. The other possibility is to distinguish the skating intensity by acceleration values, where our results would preliminarily suggest the ranges of ≥ 3, 2–3, 1–2 m/s^2^ for very high, high, and moderate accelerations, respectively. However, this separation might still have limits in the assessment of acceleration at higher velocities, where additional acceleration at high velocity requires high intensity effort. A previous study [87] used 2 m/s^2^ acceleration as the criterion for intensive skating, which might mean moderate effort while starting from stationary position or very high effort when already skating at a speed over 17 km/h. Therefore, the validation of skating intensity should be done in combination with velocity and the additional acceleration each stride.

The main limitation of this study is in the use of average acceleration and velocity which is influenced by the testing distance and the inconsistent ages and performance levels. A previous study that evaluated skating performance and age showing that sprint time did not differ between 18–30 year old players, however, was lower in players over 30 [88]. Since there were incomparable standards across selected studies, we were not able to rank participants performance levels, however the selected distance ranges a reference values should be able to distinguish performance differences as one the testing purpose. The photocell method is unable to distinguish vertical, side to side and forward acceleration and velocity, which do differ by performance level [10], and might be resolved by using accelerometers or 3D kinematics. Future research should avoid these limitations by using the same distance and reporting the skating efficiency by referring to the skating stride number, frequency, or skating efficiency coefficient. This study finds that ice-hockey testing is less reported in females, which should be amended in future research.

## CONCLUSIONS

Forward skating sprint speed increases with the measured distance up to 26 m and do not differ much from longer distance tests, while acceleration decreases with a drop below 3 m/s at distance longer 15 m and longer. The highest acceleration (5.89 m/s^2^ peak, 3.31 m/s^2^ average) was achieved in the shortest distances up to 7 m which significantly differs from 8–14 m tests. Since 6.1 m is the most often reported acceleration distance, we recommend this as an apropriate testing reference. The forward skating speed reaches its peak between 25–39 m, where 30 m is the most often reported distance and is recommended as the most practical standard for game situations and the ability to develop near maximal speed quickly. Speed measures for longest distances 40–48 m are unnecessary because 30 or 35 m provide similar or possibly higher speed values than longer distances. Future research should validate accelerometer and LPS system values to the recommended 6.1 m acceleration and 30 m sprint distance testing in all ice-hockey levels.

## References

[1] Douglas AS, Kennedy CR. Tracking In-Match Movement Demands Using Local Positioning System in World-Class Men’s Ice Hockey. J Strength Cond Res. 2020; 34:639–46. 31855927 10.1519/JSC.0000000000003414

[2] Schulze S, Laudner KG, Delank K-S, Brill R, Schwesig R. Reference data by player position for an ice hockey-specific complex test. Applied Sciences. 2021; 11:280.

[3] Farlinger CM, Kruisselbrink LD, Fowles JR. Relationships to skating performance in competitive hockey players. J Strength Cond Res. 2007; 21:915–22. 17685681 10.1519/R-19155.1

[4] Vigh-Larsen JF, Haverinen MT, Panduro J, Ermidis G, Andersen TB, Overgaard K, et al. On-Ice and Off-Ice Fitness Profiles of Elite and U20 Male Ice Hockey Players of Two Different National Standards. J Strength Cond Res. 2020; 34:3369–76. 33009345 10.1519/JSC.0000000000003836

[5] Vigh-Larsen JF, Beck JH, Daasbjerg A, Knudsen CB, Kvorning T, Overgaard K, et al. Fitness Characteristics of Elite and Subelite Male Ice Hockey Players: A Cross-Sectional Study. J Strength Cond Res. 2019; 33:2352–60. 31343551 10.1519/JSC.0000000000003285

[6] Vigh-Larsen JF, Ermidis G, Rago V, Randers MB, Fransson D, Nielsen JL, et al. Muscle Metabolism and Fatigue during Simulated Ice Hockey Match-Play in Elite Players. Med Sci Sports Exerc. 2020; 52:2162–71. 32496739 10.1249/MSS.0000000000002370

[7] Slawinski J, Termoz N, Rabita G, Guilhem G, Dorel S, Morin JB, et al. How 100-m event analyses improve our understanding of world-class men’s and women’s sprint performance. Scand J Med Sci Sports. 2017; 27:45–54. 26644061 10.1111/sms.12627

[8] Novak D, Tomasek A, Lipinska P, Stastny P. The Specificity of Motor Learning Tasks Determines the Kind of Skating Skill Development in Older School-Age Children. Sports (Basel). 2020; 8. 10.3390/sports8090126 PMC7552761 32937807

[9] Secomb JL, Dascombe BJ, Nimphius S. Importance of Joint Angle-Specific Hip Strength for Skating Performance in Semiprofessional Ice Hockey Athletes. J Strength Cond Res. 2021; 35:2599–603. 34431485 10.1519/JSC.0000000000004087

[10] Renaud PJ, Robbins SMK, Dixon PC, Shell JR, Turcotte RA, Pearsall DJ. Ice hockey skate starts: a comparison of high and low calibre skaters. Sports Engineering. 2017; 20:255–66.

[11] Shell JR, Robbins SMK, Dixon PC, Renaud PJ, Turcotte RA, Wu T, et al. Skating start propulsion: three-dimensional kinematic analysis of elite male and female ice hockey players. Sports Biomech. 2017; 16:313–24. 28534433 10.1080/14763141.2017.1306095

[12] Matthews MJ, Comfort P, Crebin R. Complex training in ice hockey: The effects of a heavy resisted sprint on subsequent ice-hockey sprint performance. J Strength Cond Res. 2010; 24:2883–7. 20940636 10.1519/JSC.0b013e3181e7253c

[13] Gilenstam KM, Thorsen K, Henriksson-Larsén KB. Physiological correlates of skating performance in women’s and men’s ice hockey. J Strength Cond Res. 2011; 25:2133–42. 21785292 10.1519/JSC.0b013e3181ecd072

[14] Buckeridge E, LeVangie MC, Stetter B, Nigg SR, Nigg BM. An on-ice measurement approach to analyse the biomechanics of ice hockey skating. PLoS One. 2015; 10:e0127324. 25973775 10.1371/journal.pone.0127324 PMC4431820

[15] Budarick AR, Shell JR, Robbins SMK, Wu T, Renaud PJ, Pearsall DJ. Ice hockey skating sprints: run to glide mechanics of high calibre male and female athletes. Sports Biomech. 2020; 19:601–17. 30200818 10.1080/14763141.2018.1503323

[16] Samozino P, Rabita G, Dorel S, Slawinski J, Peyrot N, Saez de Villarreal E, et al. A simple method for measuring power, force, velocity properties, and mechanical effectiveness in sprint running. Scand J Med Sci Sports. 2016; 26:648–58. 25996964 10.1111/sms.12490

[17] Perez J, Guilhem G, Hager R, Brocherie F. Mechanical determinants of forward skating sprint inferred from off- and on-ice force-velocity evaluations in elite female ice hockey players. Eur J Sport Sci. 2021; 21:192–203. 32241241 10.1080/17461391.2020.1751304

[18] Perez J, Guilhem G, Brocherie F. Reliability of the force-velocity-power variables during ice hockey sprint acceleration. Sports Biomech. 2022; 21:56–70. 31464169 10.1080/14763141.2019.1648541

[19] Perez J, Guilhem G, Brocherie F. Ice Hockey Forward Skating Force-Velocity Profiling Using Single Unloaded vs. Multiple Loaded Methods. J Strength Cond Res. 2021. 10.1519/JSC.0000000000004078 34175878

[20] Federolf PA, Mills R, Nigg B. Ice friction of flared ice hockey skate blades. J Sports Sci. 2008; 26:1201–8. 18608838 10.1080/02640410802027360

[21] Douglas AS, Kennedy CR. Tracking In-Match Movement Demands Using Local Positioning System in World-Class Men’s Ice Hockey. J Strength Cond Res. 2020; 34:639–46. 31855927 10.1519/JSC.0000000000003414

[22] Lignell E, Fransson D, Krustrup P, Mohr M. Analysis of High-Intensity Skating in Top-Class Ice Hockey Match-Play in Relation to Training Status and Muscle Damage. J Strength Cond Res. 2018; 32:1303–10. 28557852 10.1519/JSC.0000000000001999

[23] Douglas AS, Rotondi MA, Baker J, Jamnik VK, Macpherson AK. A Comparison of On-Ice External Load Measures Between Subelite and Elite Female Ice Hockey Players. J Strength Cond Res. 2020. 10.1519/JSC.0000000000003771 32796414

[24] Upjohn T, Turcotte R, Pearsall DJ, Loh J. Three-dimensional kinematics of the lower limbs during forward ice hockey skating. Sports Biomech. 2008; 7:206–21. 18610773 10.1080/14763140701841621

[25] Robbins SM, Renaud PJ, Pearsall DJ. Principal component analysis identifies differences in ice hockey skating stride between high- and low-calibre players. Sports Biomech. 2021; 20:131–49. 30411998 10.1080/14763141.2018.1524510

[26] Lamoureux NR, Tomkinson GR, Peterson BJ, Fitzgerald JS. Relationship Between Skating Economy and Performance During a Repeated-Shift Test in Elite and Subelite Ice Hockey Players. J Strength Cond Res. 2018; 32:1109–13. 29324580 10.1519/JSC.0000000000002418

[27] Robert-Lachaine X, Turcotte RA, Dixon PC, Pearsall DJ. Impact of hockey skate design on ankle motion and force production. Sports Engineering. 2012; 15:197–206.

[28] Allisse M, Bui HT, Desjardins P, Léger L, Comtois AS, Leone M. Assessment of On-Ice Oxygen Cost of Skating Performance in Elite Youth Ice Hockey Players. J Strength Cond Res. 2019. 10.1519/JSC.0000000000003324 31809459

[29] Ardern CL, Büttner F, Andrade R, Weir A, Ashe MC, Holden S, et al. Implementing the 27 PRISMA 2020 Statement items for systematic reviews in the sport and exercise medicine, musculoskeletal rehabilitation and sports science fields: the PERSiST (implementing Prisma in Exercise, Rehabilitation, Sport medicine and SporTs science) guidance. Br J Sports Med. 2022; 56:175–95. 34625401 10.1136/bjsports-2021-103987 PMC8862073

[30] Rico-González M, Pino-Ortega J, Clemente FM, Arcos AL. Guidelines for performing systematic reviews in sports science. Biol Sport. 2022; 39:463–71. 35309539 10.5114/biolsport.2022.106386 PMC8919872

[31] Eisenhauer JG. Meta-analysis and mega-analysis: A simple introduction. Teaching Statistics. 2021; 43:21–7.

[32] Nigg CR, Gessner A, Nigg C, Giurgiu M, Neumann R. Demographic, physiological, psychological, and on-ice performance indicators predict plus/minus status of recreational ice hockey players across a season. German Journal of Exercise and Sport Research. 2020; 50:463–9.

[33] Allisse M, Sercia P, Comtois AS, Leone M. Morphological, Physiological and Skating Performance Profiles of Male Age-Group Elite Ice Hockey Players. J Hum Kinet. 2017; 58:87–97. 28828080 10.1515/hukin-2017-0085 PMC5548157

[34] Bond CW, Bennett TW, Noonan BC. Evaluation of Skating Top Speed, Acceleration, and Multiple Repeated Sprint Speed Ice Hockey Performance Tests. J Strength Cond Res. 2018; 32:2273–83. 29878985 10.1519/JSC.0000000000002644

[35] Bracko MR, Fellingham GW. Comparison of physical performance characteristics of female and male ice hockey players. Pediatr Exerc Sci. 2001; 13:26–34.

[36] Bracko MR. On-ice performance characteristics of elite and non-elite women’s ice hockey players. J Strength Cond Res. 2001; 15:42–7. 11708705

[37] Bracko MR, George JD. Prediction of ice skating performance with off-ice testing in women’s ice hockey players. J Strength Cond Res. 2001; 15:116–22. 11708693

[38] Czeck MA, Roelofs EJ, Dietz C, Bosch TA, Dengel DR. Body Composition and On-Ice Skate Times for National Collegiate Athletic Association Division I Collegiate Male and Female Ice Hockey Athletes. J Strength Cond Res. 2022; 36:187–92. 34941612 10.1519/JSC.0000000000004175

[39] Dæhlin TE, Haugen OC, Haugerud S, Hollan I, Raastad T, Rønnestad BR. Improvement of Ice Hockey Players’ On-Ice Sprint With Combined Plyometric and Strength Training. Int J Sports Physiol Perform. 2017; 12:893–900. 27918670 10.1123/ijspp.2016-0262

[40] Delisle-Houde P, Chiarlitti NA, Reid RER, Andersen RE. Predicting On-Ice Skating Using Laboratory- and Field-Based Assessments in College Ice Hockey Players. Int J Sports Physiol Perform. 2019:1184–9. 30840516 10.1123/ijspp.2018-0708

[41] Haukali E, Lief IT. Relationship between off-season changes in power and in-season changes in skating speed in young ice hockey players. International Journal of Applied Sports Sciences. 2016; 28:111–22.

[42] Haukali E, Tjelta LI. Correlation between “off-ice” variables and skating performance among young male ice hockey players. International Journal of Applied Sports Sciences. 2015; 27:26–32.

[43] Farlinger CM, Fowles JR. The effect of sequence of skating-specific training on skating performance. Int J Sports Physiol Perform. 2008; 3:185–98. 19208927 10.1123/ijspp.3.2.185

[44] Geithner CA, Lee AM, Bracko MR. Physical and performance differences among forwards, defensemen, and goalies in elite women’s ice hockey. J Strength Cond Res. 2006; 20:500–5. 16977704 10.1519/17375.1

[45] Gupta S, Baron J, Bieniec A, Swinarew A, Stanula A. Relationship between vertical jump tests and ice-skating performance in junior Polish ice hockey players. Biol Sport. 2022:225–32. 36636195 10.5114/biolsport.2023.112972 PMC9806740

[46] Hajek F, Keller M, Taube W, von Duvillard SP, Bell JW, Wagner H. Testing-Specific Skating Performance in Ice Hockey. J Strength Cond Res. 2021; 35:S70–s5. 32149873 10.1519/JSC.0000000000003475

[47] Janot JM, Beltz NM, Dalleck LD. Multiple Off-Ice Performance Variables Predict On-Ice Skating Performance in Male and Female Division III Ice Hockey Players. J Sports Sci Med. 2015; 14:522–9. 26336338 PMC4541115

[48] Janot JM, Auner KA, Emberts TM, Kaatz RM, Matteson KM, Muller EA, et al. The effects of BungeeSkate training on measures of on-ice acceleration and speed. Int J Sports Physiol Perform. 2013; 8:419–27. 23237915 10.1123/ijspp.8.4.419

[49] Jones AM, Atter T, Georg KP. Oral creatine supplementation improves multiple sprint performance in elite ice-hockey players. J Sports Med Phys Fitness. 1999; 39:189–96. 10573659

[50] Kaartinen S, Venojärvi M, Lesch KJ, Tikkanen H, Vartiainen P, Stenroth L. Lower limb muscle activation patterns in ice-hockey skating and associations with skating speed. Sports Biomech. 2021:1–16. 10.1080/14763141.2021.2014551 34930101

[51] Kinnunen JV, Piitulainen H, Piirainen JM. Neuromuscular Adaptations to Short-Term High-Intensity Interval Training in Female Ice-Hockey Players. J Strength Cond Res. 2019; 33:479–85. 28277422 10.1519/JSC.0000000000001881

[52] Lagrange S, Ferland PM, Leone M, Comtois AS. Contrast Training Generates Post-Activation Potentiation and Improves Repeated Sprint Ability in Elite Ice Hockey Players. Int J Exerc Sci. 2020; 13:183–96. 32148640 10.70252/ONUV8208 PMC7039519

[53] Martini G, Brunelle J, Trudeau F, Lemoyne J. Measuring ice hockey skills in a repeated measures testing context: The effects of fatigue on skating efficiency, passing, agility, and shooting. Sport J. 2018; 21:1–16.

[54] Madden RF, Erdman KA, Shearer J, Spriet LL, Ferber R, Kolstad AT, et al. Effects of Caffeine on Exertion, Skill Performance, and Physicality in Ice Hockey. Int J Sports Physiol Perform. 2019; 14:1422–9. 30958066 10.1123/ijspp.2019-0130

[55] Naimo MA, de Souza EO, Wilson JM, Carpenter AL, Gilchrist P, Lowery RP, et al. High-intensity interval training has positive effects on performance in ice hockey players. Int J Sports Med. 2015; 36:61–6. 25329432 10.1055/s-0034-1382054

[56] Matthews MJ, Comfort P, Crebin R. Complex training in ice hockey: the effects of a heavy resisted sprint on subsequent ice-hockey sprint performance. J Strength Cond Res. 2010; 24:2883–7. 20940636 10.1519/JSC.0b013e3181e7253c

[57] Novák D, Lipinska P, Roczniok R, Spieszny M, Stastny P. Off-Ice Agility Provide Motor Transfer to On-Ice Skating Performance and Agility in Adolescent Ice Hockey Players. J Sports Sci Med. 2019; 18:680–94. 31827353 PMC6873137

[58] Peterson BJ, Fitzgerald JS, Dietz CC, Ziegler KS, Ingraham SJ, Baker SE, et al. Division I Hockey Players Generate More Power Than Division III Players During on- and Off-Ice Performance Tests. J Strength Cond Res. 2015; 29:1191–6. 25436625 10.1519/JSC.0000000000000754

[59] Peterson BJ, Fitzgerald JS, Dietz CC, Ziegler KS, Baker SE, Snyder EM. Off-Ice Anaerobic Power Does Not Predict On-Ice Repeated Shift Performance in Hockey. J Strength Cond Res. 2016; 30:2375–81. 26808844 10.1519/JSC.0000000000001341

[60] Perez J, Guilhem G, Brocherie F. Reliability of the force-velocity-power variables during ice hockey sprint acceleration. Sports Biomech. 2022; 21:56–70. 31464169 10.1080/14763141.2019.1648541

[61] Roczniok R, Stanula A, Maszczyk A, Mostowik A, Kowalczyk M, Fidos-Czuba O, et al. Physiological, physical and on-ice performance criteria for selection of elite ice hockey teams. Biol Sport. 2016; 33:43–8. 26985133 10.5604/20831862.1180175 PMC4786585

[62] Roczniok R, Stanula A, Gabryś T, Szmatlan-Gabryś U, Gołaś A, Stastny P. Physical fitness and performance of polish ice-hockey players competing at different sports levels. J Hum Kinet. 2016; 51:201–8. 28149383 10.1515/hukin-2015-0165 PMC5260545

[63] Roczniok R, Maszczyk A, Czuba M, Stanula A, Pietraszewski P, Gabryś T. The predictive value of on-ice special tests in relation to various indexes of aerobic and anaerobic capacity in ice hockey players. Human Movement. 2012; 13:28–32.

[64] Rønnestad BR, Slettaløkken G, Ellefsen S. Adding whole body vibration to preconditioning exercise increases subsequent on-ice sprint performance in ice-hockey players. J Strength Cond Res. 2016; 30:1021–6. 27447016

[65] Runner AR, Lehnhard RA, Butterfield SA, Tu S, O’Neill T. Predictors of Speed Using Off-Ice Measures of College Hockey Players. J Strength Cond Res. 2016; 30:1626–32. 25719922 10.1519/JSC.0000000000000911

[66] Shell JR, Robbins SMK, Dixon PC, Renaud PJ, Turcotte RA, Wu T, et al. Skating start propulsion: three-dimensional kinematic analysis of elite male and female ice hockey players. Sports Biomech. 2017; 16:313–24. 28534433 10.1080/14763141.2017.1306095

[67] Schulze S, Laudner KG, Delank K-S, Brill R, Schwesig R. Reference data by player position for an ice hockey-specific complex test. Applied Sciences (Switzerland). 2021; 11:1–14.

[68] Schwesig R, Lauenroth A, Schulze S, Laudner KG, Bartels T, Delank KS, et al. Reliability of an ice hockey-specific complex test. Sportverletz Sportschaden. 2018; 32:196–203. 30176694 10.1055/a-0648-8874

[69] Schwesig R, Hermassi S, Edelmann S, Thorhauer U, Schulze S, Fieseler G, et al. Relationship between ice hockey-specific complex test and maximal strength, aerobic capacity and postural regulation in professional players. J Sports Med Phys Fitness. 2017; 57:1415–23. 28139111 10.23736/S0022-4707.17.07020-7

[70] Schwesig R, Laudner KG, Delank KS, Brill R, Schulze S. Relationship between ice hockey-specific complex test (IHCT) and match performance. Applied Sciences (Switzerland). 2021; 11.

[71] Stenroth L, Vartiainen P, Karjalainen PA. Force-velocity profiling in ice hockey skating: reliability and validity of a simple, low-cost field method. Sports Biomech. 2020:1–16. 10.1080/14763141.2020.1770321 32546104

[72] Stetter BJ, Buckeridge E, Nigg SR, Sell S, Stein T. Towards a wearable monitoring tool for in-field ice hockey skating performance analysis. Eur J Sport Sci. 2019; 19:893–901. 30606093 10.1080/17461391.2018.1563634

[73] Thompson KMA, Safadie A, Ford J, Burr JF. Off-Ice Resisted Sprints Best Predict All-Out Skating Performance in Varsity Hockey Players. J Strength Cond Res. 2020. 10.1519/JSC.0000000000003861 33136771

[74] Stanula A, Roczniok R, Maszczyk A, Pietraszewski P, Zając A. The role of aerobic capacity in high-intensity intermittent efforts in ice-hockey. Biol Sport. 2014; 31:193–9. 25177097 10.5604/20831862.1111437 PMC4135063

[75] Stanula A, Gupta S, Baron J, Bieniec A, Tomik R, Gabrys T, et al. A Comparative Study of Two-Minute versus Three-Minute Passive Recovery on Sprint Skating Performance of Ice Hockey Forwards and Defensemen. Int J Environ Res Public Health. 2021; 18. 10.3390/ijerph182413029 PMC8701228 34948639

[76] Vigh-Larsen JF, Haverinen MT, Knudsen CB, Daasbjerg A, Beck JH, Overgaard K, et al. The relationship between age and fitness profiles in elite male ice hockey players. J Sports Med Phys Fitness. 2021; 61:512–8. 32880136 10.23736/S0022-4707.20.11313-6

[77] Wagner H, Abplanalp M, von Duvillard SP, Bell JW, Taube W, Keller M. The relationship between on-ice and off-ice performance in elite male adolescent ice hockey players—an observation study. Applied Sciences (Switzerland). 2021; 11.

[78] Williams M, Grau S. Physical Performance and the Relationship to Game Performance in Elite Adolescent Ice Hockey. International Journal of Strength and Conditioning. 2020; 1.

[79] Wilson K, Jackson J, Snydmiller G, Bell G. Development and reliability of a 7 × 15 m repeated on-ice sprint test for female ice hockey players. Int J Exerc Sci. 2021; 14:666–76. 34567374 10.70252/RPCO2660 PMC8439675

[80] Van Iterson EH, Fitzgerald JS, Dietz CC, Snyder EM, Peterson BJ. Reliability of Triaxial Accelerometry for Measuring Load in Men’s Collegiate Ice Hockey. J Strength Cond Res. 2017; 31:1305–12. 27548782 10.1519/JSC.0000000000001611

[81] Bracko MR, Fellingham GW, Hall LT, Fisher AG, Cryer W. Performance skating characteristics of professional ice hockey forwards. Sports Medicine, Training and Rehabilitation. 1998; 8:251–63.

[82] Fortier A, Turcotte RA, Pearsall DJ. Skating mechanics of change-of-direction manoeuvres in ice hockey players. Sports Biomech. 2014; 13:341–50. 25419626 10.1080/14763141.2014.981852

[83] Schulte O, Khademi M, Gholami S, Zhao Z, Javan M, Desaulniers P. A Markov Game model for valuing actions, locations, and team performance in ice hockey. Data Mining and Knowledge Discovery. 2017;31:1735–57.

[84] Stetter BJ, Buckeridge E, von Tscharner V, Nigg SR, Nigg BM. A Novel Approach to Determine Strides, Ice Contact, and Swing Phases During Ice Hockey Skating Using a Single Accelerometer. J Appl Biomech. 2016; 32:101–6. 26398967 10.1123/jab.2014-0245

[85] Vigh-Larsen JF, Haverinen MT, Panduro J, Ermidis G, Andersen TB, Overgaard K, et al. On-Ice and Off-Ice Fitness Profiles of Elite and U20 Male Ice Hockey Players of Two Different National Standards. J Strength Cond Res. 2020; 34:3369–76. 33009345 10.1519/JSC.0000000000003836

[86] Huard Pelletier V, Glaude-Roy J, Daigle AP, Brunelle JF, Bissonnette A, Lemoyne J. Associations between Testing and Game Performance in Ice Hockey: A Scoping Review. Sports (Basel). 2021; 9. 10.3390/sports9090117 PMC8473052 34564322

[87] Rago V, Muschinsky A, Deylami K, Mohr M, Vigh-Larsen JF. Weekly Training Load in Elite Male Ice Hockey: Practice Versus Competition Demands. Int J Sports Physiol Perform. 2021:1–8. 10.1123/ijspp.2021-0188 34686613

[88] Vigh-Larsen JF, Haverinen MT, Knudsen CB, Daasbjerg A, Beck JH, Overgaard K, et al. The relationship between age and fitness profiles in elite male ice hockey players. Journal of Sports Medicine and Physical Fitness. 2021; 61:512–8. 32880136 10.23736/S0022-4707.20.11313-6

